# Programmable electron-induced color router array

**DOI:** 10.1038/s41377-024-01712-x

**Published:** 2025-03-05

**Authors:** Cheng Chi, Zhibo Dang, Yongqi Liu, Yuwei Wang, Dewen Cheng, Zheyu Fang, Yongtian Wang

**Affiliations:** 1 Beijing Engineering Research Center of Mixed Reality and Advanced Display, School of Optics and Photonics, Beijing Institute of Technology, Beijing, China; 2 School of Physics, State Key Lab for Mesoscopic Physics, Academy for Advanced Interdisciplinary Studies, Collaborative Innovation Center of Quantum Matter, and Nano-optoelectronics Frontier Center of Ministry of Education, Peking University, Beijing, China; 3 College of Electrical and Information Engineering, Hunan University, Changsha, China

**Keywords:** Nanophotonics and plasmonics, Optoelectronic devices and components, Displays, Photonic devices, Sub-wavelength optics

## Abstract

The development of color routers (CRs) realizes the splitting of dichromatic components, contributing to the modulation of photon momentum that acts as the information carrier for optical information technology on the frequency and spatial domains. However, CRs with optical stimulation lack active control of photon momentum at deep subwavelength scale because of the optical diffraction limit. Here, we experimentally demonstrate an active manipulation of dichromatic photon momentum at a deep subwavelength scale via electron-induced CRs, where the CRs radiation patterns are manipulated by steering the electron impact position within 60 nm in a single nanoantenna unit. Moreover, an encrypted display device based on programmable modulation of the CR array is designed and implemented. This approach with enhanced security, large information capacity, and high-level integration at a deep subwavelength scale may find applications in photonic devices and emerging areas in quantum information technologies.

## Abstract (web summary)

We demonstrate an active manipulation of dichromatic photon momentum at a deep subwavelength scale via electron-induced CRs, and design an encrypted display device based on programmable modulation of CR array.

## Graphical abstract

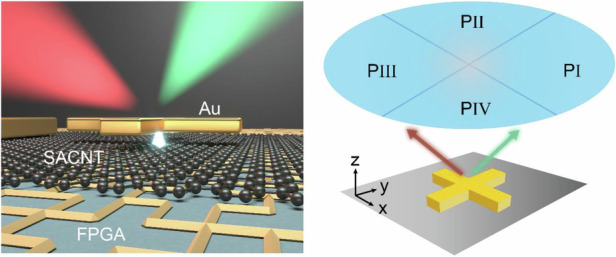

## Introduction

Steering photon propagation, with the active manipulation of optical fields and high processing speed, has been successfully applied in modern signal transmission, imaging, memory, cryptography, etc*.*^1–4^. The evolution of photon propagation science to a practical technology promises extreme advantages for specific applications in integrated optical circuits^5–7^, nano-antennas^8–12^, nano-lasers^13–15^, etc. In the development of photon manipulation technology, it is substantial to find a proper method with features of high-level integration and a large storage capacity, to satisfy the need for information transmission and processing applications^16–19^. Facing these requirements, color science with multiple frequency channels provides a promising approach to achieving large information-encoding capacity, and steering photon momentum at nanoscale has been demonstrated to be ascendant in compact information devices^20–22^. Therefore, precise modulation of photons in both frequency and spatial domains considerably benefits optical information applications^23,24^.

The color routing effect provides a unique approach for steering photon momentum in both frequency and spatial domains with high-efficiency utilization of the spectrum^25–27^. In the investigation of propagating light wavefront modulation, color routers (CRs) split light with different frequencies into divided directions, which has been utilized in light manipulation with multi-frequency channels, such as photonic crystal waveguides, frequency-encoded quantum information processing, etc*.*^28–31^. As photons are efficient information carriers with high robustness and large capacity^32^, CRs that manipulate the photon momentum in multi-frequency channels can be applied for display and information technologies, especially optical information encoding and encryption with high dimensionality and low crosstalk. Previous studies on CRs mainly focus on structure design, where the modulation of photon momentum can be realized with metasurfaces, nanoantennas, gratings, *etc*^33–36^. A recent study reveals that CRs can be observed within single silver nanorods^37^, which is however still difficult for further on-chip applications due to lacking flexible manipulation at nanoscale. Therefore, an efficient solution in active controllable CRs needs to be proposed to realize its full potential in optical information applications.

As one of the noninvasive high-resolution detection methods, electron beam spectroscopy such as cathodoluminescence overcomes the light diffraction limit, which has been successfully applied for electromagnetic field investigation at deep subwavelength scale^38–40^. Under electron beam stimulation, the deep subwavelength shift of impact position can directly regulate the distribution of local density of states, contributing to the evolution of far-field emission patterns^41,42^. As a characterization of photon momentum, angle-resolved polarimetry can explore propagation modes of multicolor photons with far-field patterns and allows investigation of frequency-dependent spin-orbit locking with high resolution^43^. This technique overcomes the light diffraction limit and thus can be applied to steer the electron-induced CRs at deep subwavelength scale.

In this work, we demonstrate a modulation of dichromatic photon momentum via electron-induced CRs at deep subwavelength scale. The green and red components of far-field emission are routed into different propagation directions when the electron beam from the on-chip source impinges on the edge of a single Au nanoantenna, while the non-splitting pattern is observed with the impinging position located at the center. Furthermore, the conversion of the featured radiation pattern can be triggered by steering the electron beam impact position. The active modulation of dichromatic photon splitting can be effectively achieved by altering the far-field interference of dipole and quadrupole moments with judiciously adjusted impinging position. More importantly, based on this principle of electron-induced CRs, we realize a programmable encrypted display device with the CR array, which provides a compelling platform for the manipulation of photon momentum at nanoscale and paves the way for future quantum information technology and integrated photonic systems.

## Results

### On-chip electron-induced CRs

**Fig. 1:**
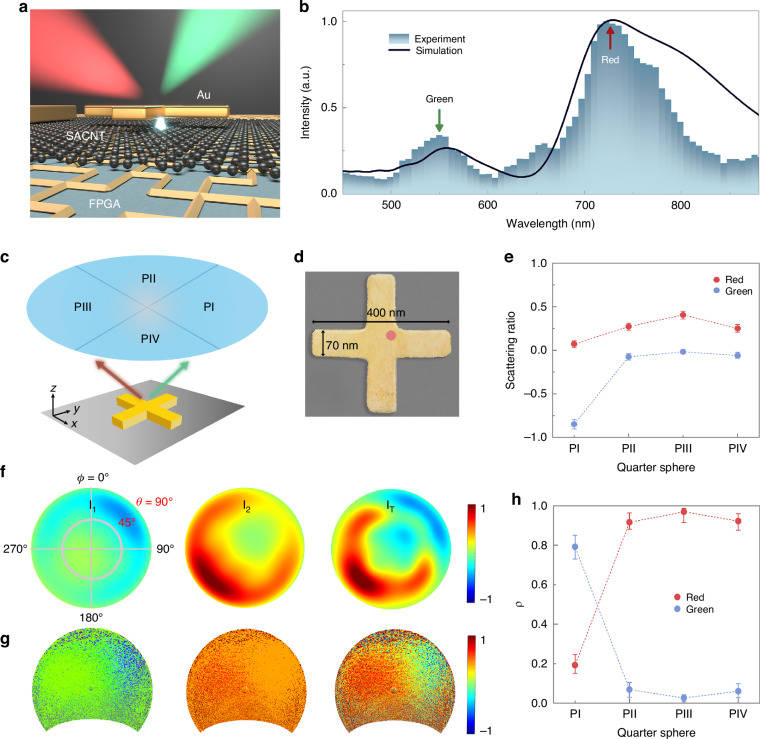
**Design of on-chip CRs and its photon momentum modulation function. a** Schematic of electron-induced CRs. Symmetrical Au nanoantenna under electron beam stimulation at the nanoantenna corner generates asymmetrical dichromatic dispersion radiation. **b** Experimental and simulated spectra obtained from Au nanoantenna with the size of 400 nm × 70 nm. The blue (black) curve corresponds to the experimental (simulated) spectrum. The peak positions of green and red components are marked with arrows. **c** Schematic of dichromatic component analyzing. Four quarters from P_I_ to P_IV_ are introduced to detect dichromatic photon propagation directions. **d** Pseudo-color scanning electron microscopy (SEM) image of a single Au nanoantenna. The stimulation position is located at the upper-right corner of the Au nanoantenna. **e** Scattering ratio of different angular quarter sphere detection, where the ratio value of the red (green) component is defined to be positive (negative) as a distinction. The error bar represents the uncertainty of the scattering ratio in multiple experiments. **f** Simulated angular S_0_ patterns of green (I_1_) components at 560 nm and red (I_2_) components at 720 nm for signals. The differential angular pattern is shown in I_T_. The nanoantenna size and stimulation position are the same as **d**. **g** Measured angular S_0_ patterns of green (left) and red (center) components, which are acquired by using different bandpass filters with a 50 nm bandwidth and differential components. The differential angular pattern is shown on the right. The axis in **g** is the same as in **f**. **h** Intensity ratio (ρ) of the red and green components in each region. The error bar represents the uncertainty of ρ, which is extracted from multiple measurements

The electron-induced CR splits the dichromatic photon momentum, where the asymmetrical radiation pattern of dichromatic photons is observed under electron stimulation (Fig. 1a). The single nanoantenna in the shape of a cross is fabricated at a size of 400 nm × 70 nm with the deposition of 60 nm Au (Fig. 1d). To investigate the far-field behavior of different color components, the dichromatic photon splitting schematic (Fig. 1c) is introduced to retrieve frequency-related quantity, where far-field measurements are performed to retrieve angular patterns of dichromatic components (Supplementary Note 1).

When the upper-right corner (Fig. 1d) is excited, the emission of the Au nanoantenna is analyzed both in the spectrum and far-field angular patterns. The spectrum shows that emission intensity peaks located at the wavelength around 560 nm (Green) and 720 nm (Red), agreeing well with the simulation result (Fig. 1b), with a slight shift compared to the local plasmonic resonance (Supplementary Fig. S1). Far-field patterns of dichromatic components are acquired with angle-resolved detection and Stokes parameter S_0_ is retrieved. To further explore the far-field behavior of the dichromatic components, the panchromatic angular pattern is investigated, and the direction splitting of the green and red components is observed (Fig. 1f, g). This result demonstrates the distinct far-field patterns of green and red components, which can be applied for the manipulation of dichromatic photon momentum. Nevertheless, probing the same sample with a diffraction-limit optical spot will wash out the CR performance (Supplementary Fig. S2), where the propagation of the green and red components doesn’t show any directionality.

To explore the directionality of green and red components under different impinging positions, the scattering ratio of each component is acquired in the four angular regions from P_I_ to P_IV_, which is defined as the proportion of monochromatic radiative power in each region among the total. The experimental data shows that the measured scattering ratio reaches beyond 80% in P_I_ for the green component, and beyond 90% integrated from P_II_ to P_IV_ for the red component (Fig. 1e). These results indicate the emission directionality of the green and red components in far-field angular patterns as shown in Fig. 1b. To quantitatively describe the intensity contrast between red and green components in different directions, the intensity ratio ρ is defined as I_α_/I_total_ (α = red or green), where I_α_(I_total_) is the integrated intensity of α component (total) in each region. Extracted from experimental results with an error bar to describe the uncertainty, Fig. 1h shows that ρ_green_ remains around 80% with a deviation below 8% in P_I_ and ρ_red_ remains beyond 90% with a deviation below 6% from P_II_ to P_IV_. These analyses not only demonstrate the directionality of monochromatic emission, but also verify the splitting pattern between red and green components.

### Principles of electron-induced CRs

**Fig. 2:**
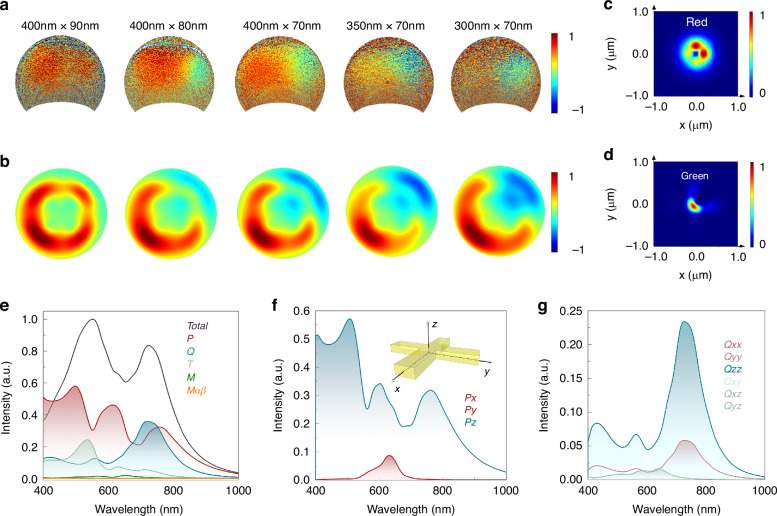
**Theoretical analyses of multipole component patterns for Au nanoantenna. a** Experimental angular dichromatic patterns obtained from Au nanoantennas at the size of 400 nm × 90 nm, 400 nm × 80 nm, 400 nm × 70 nm, 350 nm × 70 nm, and 300 nm × 70 nm. The stimulation position is located at the upper-right corner of the Au nanoantenna. **b** Simulated angular dichromatic patterns of Au nanoantenna at different sizes. The Au nanoantenna size and impinging position are the same as in **a**. **c**, **d** Simulated near-field intensity pattern of red and green components. **e** Simulated scattering intensity of various multipole moments in the nanoantenna with the size of 400 nm × 70 nm, including the total intensity and the intensities of the five most contribution moments as *P*, *Q*, *T*, *M*, *M*_*αβ*_. **f** Simulated scattering intensity of various electrical dipole components in the nanoantenna with the size of 400 nm × 70 nm, including the intensity of *Px*, *Py*, and *Pz* components. Inset: axis (*x, y, z*) of the dipole orientation. **g** Simulated scattering intensity of various electrical quadrupole components *Q*_*αβ*_ in the nanoantenna with the size of 400 nm × 70 nm

To explore the mechanism of electron-induced CRs, far-field angular patterns of different-sized Au nanoantennas are acquired with angle-resolved cathodoluminescence (CL) imaging spectroscopy (Supplementary Note 2). The bandpass filters are centered at 730 nm and 550 nm with a bandwidth of 50 nm. As the color router has a broad CL peak in the spectrum, we change the bandpass filters to the type centered at 700 nm and 580 nm (50 nm bandwidth), and the type centered at 730 nm and 550 nm (10 nm bandwidth) for comparison. Results show that the color routing effect can also be observed when the filter center shifts (Fig. S19a). As for filters with narrow bandwidth, more integral time is needed for pattern characterization on the Fourier plane (Fig. S19b). The angular patterns of the dichromatic components with the impinging position located at the upper-right corner are shown in Fig. 2a, where distinguishable splitting of the green and red components validates the existence of CRs in different nanoantennas. The splitting pattern of dichromatic components becomes most distinguishable with the Au nanoantenna size at 400 nm × 70 nm agreeing well with simulated results (Fig. 2b, Supplementary Fig. S3), which can be obtained from the inverse design (Supplementary Fig. S4). The further change in size influences the output multipolar modes, thus resulting in the far-field pattern shift. The emission intensity ratio between red and green components and corresponding angular patterns can be modulated and shifted with the length and width of the nanoantenna increase as shown in Supplementary Fig. S5. Asymmetric nanoantennas result in asymmetric mode distribution, thus providing another route in pattern modulation. The emission intensity ratio between red and green components and corresponding angular patterns can be modulated by the asymmetric nanoantennas as shown in Supplementary Fig. S6. While in the encrypted display, symmetric nanoantennas can regulate the far-field angular emission pattern by exciting symmetric position, thus making the device more practical and controllable in the application. Besides, the encrypted information in symmetric nanoantennas can only be read out by far-field pattern detection and decided by the impinging position instead of the structural shape, thus improving the encryption performance of the device. By further analyzing the calculated near-field signal of the green and red components, distinct spatial separation of their near-field patterns is observed, which result in the splitting of green and red components in the far field (Fig. 2c, d). This splitting feature indicates the performance of electron-induced CRs.

To elucidate the underlying physics of the observed CRs under electron stimulation, the scattered intensity of different multipole moments is investigated with multipolar decomposition, where the simulated radiative field with stimulation position located at the upper-right corner is decomposed into vector spherical harmonics according to the general multipole scattering theory^44^:

1 $$\begin{array}{c}I=\displaystyle\frac{2{\omega }^{4}}{3{c}^{3}}{\left|P\right|}^{2}+\frac{2{\omega }^{4}}{3{c}^{3}}{\left|M\right|}^{2}+\frac{4{\omega }^{5}}{3{c}^{5}}\left(P\cdot T\right)+\frac{2{\omega }^{6}}{3{c}^{5}}{\left|T\right|}^{2}\\ +\displaystyle\frac{{\omega }^{6}}{5{c}^{5}}\sum {\left|{Q}_{\alpha \beta }\right|}^{2}+\frac{{\omega }^{6}}{20{c}^{5}}\sum {\left|{M}_{\alpha \beta }\right|}^{2}+\frac{{\omega }^{8}}{20{c}^{7}}\sum {\left|{T}_{\alpha \beta }\right|}^{2}+O\left(\frac{1}{5{c}^{7}}\right)\end{array}$$

where *P*, *M*, *T*, *Q*_*αβ*_, *M*_*αβ*_, and *T*_*αβ*_ correspond to the electrical dipole, magnetic dipole, toroidal dipole, electrical quadrupole, magnetic quadrupole, and toroidal quadrupole respectively; *c* is the speed of light in vacuum; *α*, *β* = *x, y, z*. Considering the circumstance of the electron-beam stimulation at the corner, relevant components *P*, *Q*, and *T* are calculated with asymptotic far-field approximations for dipoles and quadrupoles in the presence of a substrate. *P* components are featured by characteristic toroid shapes with a significant *E*_*z*_ component at the corner, suitable for electron beam modulation, *Q* quadrupoles show multiple radiation lobes in the far-field pattern.

With the impinging position located at the upper-right corner of the Au nanoantenna (Fig. 1d), the scattering intensity of different multipole moments (Fig. 2e) is calculated from the finite-difference time-domain (FDTD) simulated data. At the wavelength of 560 nm, the contribution of *P, Q*, and *T* moments dominates in the far-field angular pattern, and their interference mode leverages the far-field angular pattern. Further calculation of scattering intensity for electrical dipole moments reveals that *P*_*z*_ is the major component in the far-field radiation of electric dipole at the wavelength around 560 nm (Fig. 2f) while *P*_*x*_ and *P*_*y*_ possess the same intensity due to the stimulation position on the symmetry axis. At the wavelength of 720 nm, the scattering intensity of multipole moments shows that *P*_*z*_ and *Q*_*αα*_ (*Q*_*xx*_, *Q*_*yy*_, *Q*_*zz*_) (Fig. 2g) are major components in the far-field radiation. Therefore, the contribution of interference modes is dominant in the far-field angular pattern and results in the splitting pattern of dichromatic components. With the impinging position shifting to the lower-left corner, the phase difference of π/2 between quadrupole and dipole moments results in the inversion of the splitting pattern between dichromatic components. The size difference of Au nanoantennas contributes to the varied proportion of multipole moments, where the proportion difference between *P* and *Q* moments promotes as the size of nanoantennas increases, thus resulting in the far-field splitting pattern evolution of dichromatic components. Further we calculated the intensity of *M* and *M*_*αβ*_ components as shown in Fig. 2e, which has less contribution to the total intensity at the wavelength around 560 nm and 720 nm compared to *P*, *Q* and *T*. The high orders related to coupling among *T, M*_*αβ*_ and *T*_*αβ*_ contribute less to the resonance because of the weak intensities of *M*_*αβ*_ and *T*_*αβ*_ $({I}_{{M}_{\alpha \beta }} > {I}_{{T}_{\alpha \beta }})$. These couplings are associated with the analysis of polarization transformation^45,46^, which has less effect on the design symmetry nanoantennas in the color routers.

The underlying physical basis for the electromagnetic mode imaging is the reciprocity relation between electron beam excitation and optical plane wave illumination. By employing the Lorentz reciprocity theorem, it is demonstrated that the electron beam-generated cathodoluminescence *E*_1_ can be directly related to the normally incident optical plane wave-induced electric field *E*_2_ via the relation^47^:

2 $${E}_{1}\left({{\bf{r}}}_{0},\omega \right)=\frac{i\omega e}{4\pi {\varepsilon }_{0}{c}^{2}R}{\text{e}}^{-i\omega t}{\int }_{\!\!-{{\infty }}}^{{{\infty }}}{E}_{2}\left({x}_{0},{y}_{0},z,\omega \right){{\bf{n}}}_{z}{\text{e}}^{-i\omega z/v}{dz}$$

The cathodoluminescence emission is related to the integral of the *z* component of *E*_2_(**r**, ω) with specific phase e^−^^*iωz/v*^, reflecting the electric field intensity distribution along the *z* direction under plane wave excitation. The electron excitation could precisely manipulate the impinging position at the nanoscale, thus realizing the selective excitation of specific multipolar modes and modulating their phase relation. Further, the electron excitation can excite some high-order modes that can hardly be excited by the plane wave.

### Sub-wavelength modulation of electron-induced CRs

**Fig. 3:**
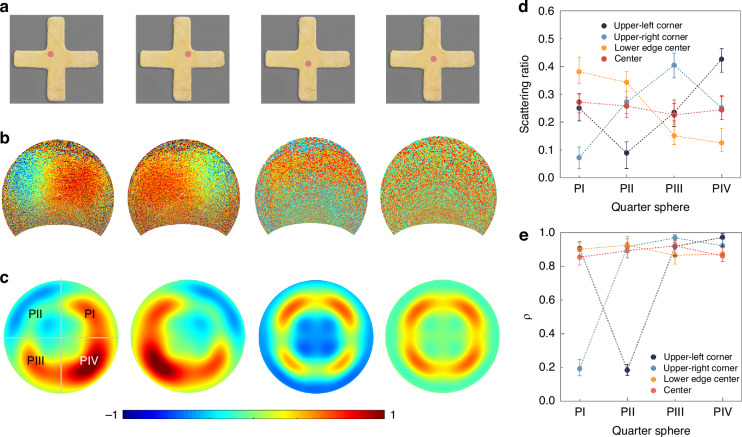
**Manipulation of dichromatic photon momentum. a**, **b** Experimental angular dichromatic patterns obtained from Au nanoantenna at the size of 400 nm × 70 nm. Stimulation positions are located at the upper-left corner, upper-right corner, center of the lower edge, and center of the nanoantenna, which are marked by red points on the pseudo-color SEM image of the Au nanoantenna as shown in **a**. **c** Simulated angular patterns of the Au nanoantenna. Electron beam stimulation positions are the same as in **a**. **d** Scattering ratio of the red component in different region detection. Electron beam excitation positions are the same as in **a**. **e** Intensity ratio (ρ) of the red component with different region detection. The definition of intensity ratio (ρ) is the same as in Fig. 1h. Electron beam impinging positions are the same as in **a**

Programmable modulation of dichromatic photon momentum at deep subwavelength scale by electron-induced CR device is shown in Fig. 3. Four impinging positions are chosen to illustrate this manipulation at detected wavelengths of 560 and 720 nm, respectively (Fig. 3a). For the impact position at the upper-left corner, the far-field angular pattern shows photon splitting with green component located at the P_II_ region and red component located at rest three regions of the angular hemisphere, which demonstrates a turn-on state of CR (Fig. 3b). For the stimulation position located at the middle of the edge or the center of the nanoantenna, the splitting between green and red components is not observed, which shows a regression to the turn-off state of CR. For the impinging position located at the upper-right corner, the relative location of green and red components converts in the splitting pattern, which manifests returning to the turn-on state. The switch from the turn-on to the turn-off state can be achieved with the impinging position shift within 30 nm, and the conversion of the angular pattern can be realized within 60 nm, demonstrating an efficient active manipulation of dichromatic photon momentum by electron-induced CR. As shown in Fig. 3c, simulated results reveal the angular pattern evolution with impact positions at four chosen positions, which is consistent with the experimental results. For impinging positions with more deviation from the corner, simulated results (Supplementary Fig. S7) show CR with diminished splitting. These results indicate that the distinguishability of the dichromatic component splitting in the far field reaches its maximum at the corner. Various excitation positions ignite different multipole modes, resulting in the peak shift in the spectrum^38^. Three different impinging positions are selected with the spectrum measured as shown in Supplementary Fig. S8, where peak shift is observed with the excitation position changed.

To quantitively describe the directionality of dichromatic components under different impinging positions, the scattering ratio of the red component is acquired with different impinging positions. The experimental data show that the measured scattering ratio of the red component stays below 10% for only one region when excited at the upper-left or upper-right corner (Fig. 3d), which indicates the emission directionality in the far-field angular pattern. However, when the impinging position shifts to the center of the nanoantenna, the emission directionality of the red component disappears. In addition, to investigate the intensity contrast between dichromatic components, the intensity ratio ρ of the red component in each region is illustrated in Fig. 3e, showing that ρ stays below 20% with a deviation less than 5% in region P_II_ (P_I_) when the stimulation position is located at the upper-left (upper-right) corner. These analyses demonstrate the different splitting patterns of dichromatic components under various impinging positions, verifying the steering of electron-induced CRs via shifting the stimulation position at deep subwavelength scale, by which the active manipulation of the dichromatic photon momentum is achieved. With the structure size shift, the intensity ratio of red and green components can be further manipulated (Supplementary Fig. S9).

**Fig. 4:**
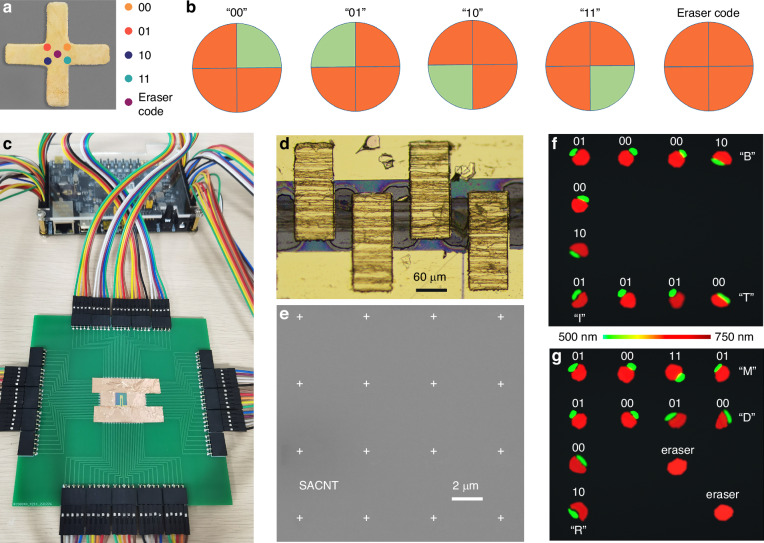
**Schematic and performance of frequency-dependent encrypted display. a** Functional unit for frequency-dependent quaternary encoding. With electron beam impinging positions located at four inner corners and the center of the nanoantenna, which are marked with five points on the pseudo-color SEM image; corresponding color routing patterns are defined as 00, 01, 10, 11, and the erasure code for quaternary encoding. **b** Angular patterns of the dichromatic components in quaternary encoding, displayed in the order of 00, 01, 10, 11, and erasure code. **c** Image of the CRs on the PCB board and Field-Programmable Gate Array controller. **d** Photograph of SACNT film and gold electrodes, scale bar is 60 μm. **e** The SEM image of SACNT flake and Au cross nanoantenna array fabricated by electron-beam lithography, scale bar is 2 μm. **f**, **g** Two-dimensional encrypted display of capital letters “C” and “R”. In the capital letter “C”, the measured splitting patterns of dichromatic components output “01000010”, “01001001” and “01010100” corresponding to ASCII codes of capital letters “B”, “I” and “T”, respectively. In the capital letter “R”, three character strings of “01001101”, “01010010” and “01000100” are output as capital letters “M”, “R” and “D”, respectively

In the experiment measurement of a single router, SEM images were pictured to verify the accuracy of the excitation component position to the router. The distance between nearby tips of emitting cathodes is 50 nm (Supplementary Fig. S12a), which is close to the width of the router unit at 70 nm (Fig. 4a). The detection of SEM images shows that the tips are sheltered by the nanoantenna (Fig. 4a). As the tips are aligned and patterned regularly, the SEM image verifies the accuracy of electron emission collimation. With deflection electrodes introduced to manipulate the electron’s locus, electrons could impinge on the router with a small divergence angle and little position offset. Further, in the display application experiment, the simulated far-field angular pattern of red and green components that has been verified by the proof-of-principle experiments, could act as the standard for testing the accuracy of electron emission and performance of each router. To achieve the precision at the nanoscale, the flatness of the SACNT/substrate and pattern alignment precision of nanoantennas should be well processed. Oxygen plasma at a power of 20 W is applied to clean the SACNT for 20 s. To improve the flatness of the Si/SiO_2_ substrate, oxygen plasma at a power of 75 W is applied to clean the atomically flat Si wafer for 2 minutes to avoid emission position shift. To improve the pattern precision, The SACNT is marked using electron beam lithography and e-beam evaporator deposition to create corresponding Au markers (5-nm-thick Ti and 60-nm-thick Au) on the silicon substrate, followed by a lift-off process. Then, metallic nanoantennas and contacts were patterned on SACNT by electron beam lithography with the assistance of alignment marks to realize high-precision emission, followed by thermal deposition and lift-off process.

### Programmable CR array for encrypted display

The manipulation of CRs under electron stimulation can be applied for a frequency-dependent quaternary encoding, where the dichromatic photon momentum can be manipulated by shifting the impinging position at deep subwavelength scale. The coding mode is dependent on the radiation from a single nanoantenna, where the splitting dichromatic patterns in the angular hemisphere are encoded in quaternary. An erasure code defined from the non-splitting pattern is introduced to improve the information accuracy. These five patterns defined as 00, 01, 10, 11, and erasure code correspond to five electron beam impinging positions on the upper-right, upper-left, lower-left, lower-right corner, and the center of the encoding unit (Fig. 4a), respectively. Corresponding angular patterns of the dichromatic components are shown in Fig. 4b. In this way, the whole encoding process is integrated into a single unit (Supplementary Fig. S10).

To develop a practical device for frequency-dependent encrypted display based on this encoding strategy, color routers are designed and fabricated in the shape of a 4 × 4 nanoantenna array. These encoding units are precisely patterned on an on-chip thermionic electron source array (super-aligned carbon nanotube films, SACNT), as shown in Fig. 4e. The emitting of aligned electrons can be controlled by gold electrodes (Fig. 4d). Then, the whole device is integrated on PCB board and manipulated by Field-Programmable Gate Array (FPGA) as shown in Fig. 4c and Supplementary Fig. S11. The schematic shown in Supplementary Fig. S10 realizes the switchable control of each nanoantenna unit. The manipulation of five excitation positions in each unit is realized by the excitation component beneath the SACNT film, which consists of five emitting cathodes and gate electrodes arranged compactly as shown in Supplementary Fig. S12a. Among them, Cr tips were prepared on monocrystalline Si chips. Electron beam lithography (Raith e-LINE) was applied for a single-layer nanocavity with a surrounding groove as shown in Supplementary Fig. S12b. The FPGA is integrated below the emission cathode to enable flexible tunable and fast response of the field excitation device. It controls a level-shifter to each pixel and employs vertical and horizontal scanning circuitry, as shown in Supplementary Fig. S12c. The selective excitation of different positions on the SACNT film results in electron enrichment and leverages the electron emission position towards routers with tight adhesion to SACNT at nanoscale precision. By picking a certain output port of electrons with FPGA, a high-level integration and selective stimulation is achieved.

The proposed encrypted display is programmed in a 4 × 4 position matrix. With a predefined read-out logic (Supplementary Note 3), the position matrix is set according to the 4 × 4 pixel image and the encrypted information encoded in American Standard Code for Information Interchange (ASCII). These 8-digit sequences (ASCII codes) can be divided into 2-digit units, which are assigned to four matrix elements. Then the binary code in each matrix element decides the electron impinging position in the corresponding unit cell. Connected to a computer via USB, this reconfigurable device is used to display characters where a hidden character string is decrypted in the momentum space. As a demonstration, capital letters “C” and “R” are displayed in spatially resolved photographs (Supplementary Fig. S13). To switch the display mode from color display to encrypted display, a plate of micro-lens array with a diameter of 4 μm for each unit is placed in front of the device (Supplementary Fig. S14), which reads out the encrypted information on the Fourier plane based on the emission direction analysis of green and red components. Character strings “BIT” and “MRD” are encrypted in the photograph and can only be decrypted by far-field detection on the optical Fourier surface (Fig. 4f–g), where the measured ρ in four regions meets the read-out need (Supplementary Fig. S15). Apart from capital letters, various graphics and characters can be displayed in pixels and encrypted according to ASCII. With the eraser code, characters “4+” are encrypted as shown in Supplementary Fig. S16, with a square graphic displayed.

This device utilizes dichromatic photon momentum and beam intensity as the carrier to promote information processing ability, which increases information capacity based on frequency-dependent angular measurement. The spatial distribution of active unit cells presents the designed image for display. However, the constant integrated intensity ratio between the green and red components in the whole angular space with different impinging positions prevents the encoding information from being read out by conventional intensity detection. The distinct angular patterns of dichromatic photon momentum splitting in momentum space are the key to decoding the encrypted image. The introduction of erasure code improves the robustness of the encoding process, contributing to a reliable encryption solution. This frequency-dependent encrypted display overcomes the light diffraction limit with controllable electron excitation and realizes the deep subwavelength scale leverage of dichromatic photon momentum that acts as the coding information. Furthermore, the size of the practical device including the integrated electron source array can be minimized down to micron-scale, which could bring us much closer to realizing the untapped potential of CRs for numerous applications. In the future work, higher plasmonic Q-factors materials such as Al and Ag can be introduced to the display device design with better performance, while the vacuum packaging of Al and Ag nanoantennas should be well processed to avoid potential material oxidation’s influence on the device. Besides, we fabricated Al nanoantennas at a size of around 300 nm and characterized their electron-induced spectrum features, where the measured spectrum shows a peak around 400 nm (Supplementary Fig. S17), thus providing a potential solution for modulating blue components in color routers.

## Discussion

In summary, we have demonstrated a unique approach for a programmable encrypted display via an electron-induced CR array. The switch between the “on” and “off” states of CR is realized with an impinging position shift within 30 nm, and the far-field radiation pattern conversion is manipulated by steering the impact position within 60 nm. Multipolar decomposition indicates that the multipole moments excited by electrons leverage the pattern evolution of angular splitting between red and green components. Furthermore, as photons can act as excellent information carriers for information applications, we demonstrate a programmable encrypted display device with an integrated electron source array. Features of deep subwavelength scale modulation, large information capacity, and enhanced security make it a promising candidate for information storage and processing, where high integration and minuscule size broaden its practical applications. Our work provides a demonstration of modulating photon momentum via electron-induced CR and programmable color router array for encrypted display, which can ignite modern interdisciplinary research in on-chip spectroscopy, optical communication, and related applications in integrated quantum information technology.

## Materials and methods

### Nanostructures fabrication

The nanostructures are fabricated with a standard EBL process followed by a lift-off process and Ar ion irradiation. In detail, a positive resist (MircoChem PMMA (poly (methyl methacrylate)) A4 950) is spin-coated onto the substrate with a thickness of ~60 nm. Structures are patterned by using a focused 30 keV electron beam controlled by the Nano Pattern Generation System module (Raith), which is equipped on the SEM (Zeiss Supra55). A 60 nm Au layer is deposited on the substrate by using an electron beam evaporator (HVC-800DA). Ar ion irradiation is performed for 1 min to clean up the residual PMMA in the final process.

The nanostructure templates are soaked in ethanol and cleaned by ultra-sonication in ethanol for 15 min until the films are removed and rinsed with ethanol. The masters are kept in an oven at 75 °C for 1 h. After drying, a commercial PDMS solution is cast on the template layer, followed by a degassing and curing process at 70 °C for 4 h in a vacuum oven. Then, the nanostructure-patterned PDMS film is peeled off the template. A 60 nm Au layer is deposited on the nanostructures by using an electron beam evaporator (HVC-800DA).

### On-chip electron sources fabrication

Firstly, SACNT films are transferred to a 1 cm × 1 cm SiO_2_/Si substrate. Secondly, SACNT films on the substrate are patterned to strip arrays by reactive ion etching. Thirdly, interdigital electrodes are patterned via electron beam lithography, followed by electron beam evaporation deposition (Au/Cr = 160/5 nm) and a standard lift-off process. In order to facilitate electrical measurements, a pair of contacting pads with the dimensions of 1.2 μm × 0.6 μm are fabricated for emitters. Then, SiO_2_ underneath the SACNT films and nanostructures between the electrodes is selectively etched off by buffered hydrofluoric acid to make the SACNT films and nanostructures suspended.

### Angle-resolved optical measurements

The optical measurements are performed using a paraboloid mirror (0.1 parabola coefficient, 0.5 mm focal distance, 1.46π sr acceptance angle, 10 nm RMS roughness, and λ/2 curve accuracy) and 2D back-illuminated CCD array. The mirror collects the generated optical information and redirects it to the achromatic lens, which is defocused to ensure that the beam fills the CCD array. This Fourier imaging consists of imaging the back-aperture of a microscope objective that contains the full wave vector information of emitted light onto a CCD. Each measurement is taken with a different setting of the 50 nm band-pass color filters spectrally selected for the measured emission. For every setting of the filters, we collect a dark reference measurement where we turn off the electron beam, which is subtracted from the data in the post-processing stage. Possible sources of errors in the detection include e-beam drift (in the case of position-dependent samples), bleaching/contamination during measurements leading to a reduction in the optical signal, and fluctuations in current and mirror alignment.

### Angle-resolved CL Imaging Spectroscopy

CL angular patterns are acquired by a CL detector system (SPARC), which is equipped on the SEM (Thermofisher Scientific, Quattro C). The emission is collected by a high-sensitive CMOS. For detecting specific wavelengths of the CL emission, different bandpass filters are placed in the optical path. The substrate background signal (*I*_background_) is subtracted from each pixel (*I*_raw_) in the raw CL angular pattern, and the resulting CL signals of each pixel (*I*_CL_) are corrected based on the collection efficiency of the system with the correction equation expressed as *I*_CL_ = (*I*_raw_ − *I*_background_)/Δ*λ*_3dB_/*η*_system_. Δ*λ*_3dB_ is the effective 3 dB bandwidth of each bandpass filter, and *η*_system_ is the corresponding collection efficiency including contributions of the CMOS at each center wavelength of the bandpass filter. Angular collection range is up to 1.46π sr, with angular resolution <10 mrad.

### Numerical simulations

All simulation results in this report are all accomplished by commercial finite difference time domain methods solver (FDTD Solutions, Lumerical). The simulation domain includes the structure with perfectly matched layers in all directions. For the calculation of scattering, total-field scattered-field sources with linear and circular polarization are used to illuminate the structure along the −*z* axis. In the FDTD simulation, the electron beam moving along the −*z* axis is regarded as a linear current density **J**(***r***, t) = *ρvδ(z* + *vt) δ(x* − *x*_0_) *δ(y* − *y*_0_*)****n***_z_, where *ρ* is the electron charge, *v* is the velocity of the electron, ***r*** = (*x*_0_, *y*_0_, *z*) is the position of the electron beam, and ***n***_z_ is the unit vector along the +*z* direction. The set of ***r*** = (*x*_0_, *y*_0_, *z*) controls the position of ebeam irradiation on the *x* − *y* plane in FDTD simulation. In the frequency domain, it corresponds to a current density as **J**(***r***, ω) = *ρ*e^−*iωz/v*^*δ(x* − *x*_0_) *δ(y* − *y*_0_*)****n***_z_, and then the current density is modeled as a series of dipoles with a temporal phase delay (−*z/ν*) related to the electron velocity. A reference simulation (without the nanostructure and substrate) is also run to subtract any background signal created by only the electron beam that could obscure the signal from the nanostructure. The spectra are calculated in the far field by integrating the Poynting vector normal to an arbitrary surface in the upper *z* half-plane for the wavelengths ranging from 400 to 1000 nm. The far-field region is set as a vacuum. In the simulations, we use Palik data for the Au (gold) CRC, Si (Silicon) complex refractive indices. The refractive index of SiO_2_ is taken as 1.5.

## Supplementary information

supplemental material for Programmable Electron-induced Color Router Array

## Data Availability

The datasets generated during or analyzed during the current study are available from the corresponding author on reasonable request.
