# Supplementary material for: Programmable electron-induced color router array
Source: Light Sci Appl. 2025 Mar 5;14:111. doi: 10.1038/s41377-024-01712-x (PMC11882952; doi:10.1038/s41377-024-01712-x)
Supplement: Supplementary file 1 — supplemental material for Programmable Electron-induced Color Router Array [file 41377_2024_1712_MOESM1_ESM.pdf]

Supplementary Information for

Programmable Electron-induced Colour Router Array

Cheng Chi[1#], Zhibo Dang[2#], Yongqi Liu[1#], Yuwei Wang[3], Dewen Cheng[1], Zheyu Fang[2*], Yongtian Wang[1*]*

[1]Beijing Engineering Research Center of Mixed Reality and Advanced Display, School of Optics and Photonics, Beijing Institute of Technology, Beijing 100081, China
[2]School of Physics, State Key Lab for Mesoscopic Physics, Academy for Advanced Interdisciplinary Studies, Collaborative Innovation Center of Quantum Matter, and Nano-optoelectronics Frontier Center of Ministry of Education, Peking University, Beijing 100871, China
[3]College of Electrical and Information Engineering, Hunan University, Changsha 410082, China

e-mail: cdwlxk@bit.edu.cn; zhyfang@pku.edu.cn; wyt@bit.edu.cn

This file includes:

Supplementary Figs S1 to S21
Supplementary Notes 1 to 3
References

Supplementary Text

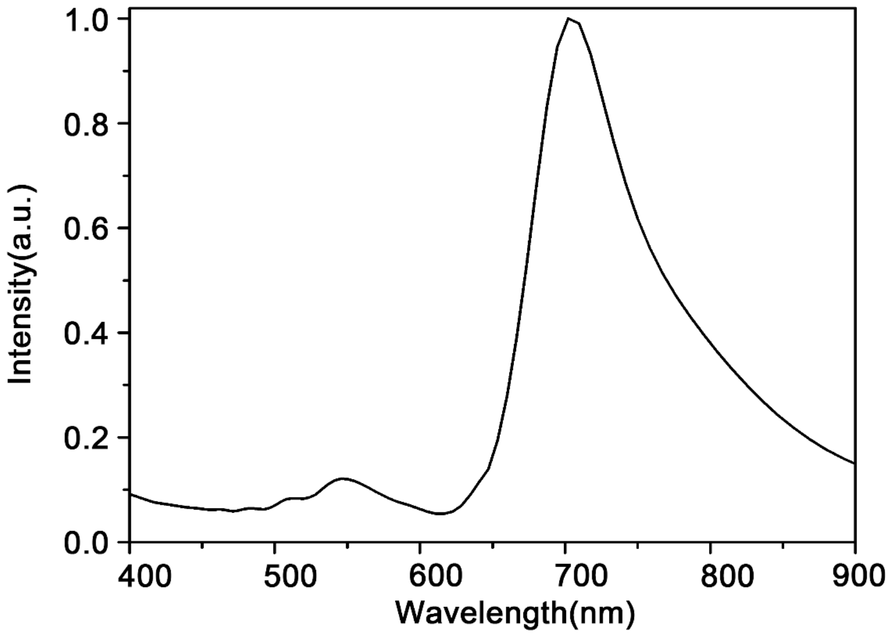

Fig. S1 Simulated scattering spectrum of Au nanoantenna. The simulated spectrum was acquired on an Au nanoantenna with the size of 400 nm×70 nm under the normal incident plane wave. The peak wavelengths are around 550 nm and 720 nm, corresponding to green and red components. The acquired wavelength range is from 400 to 900 nm.

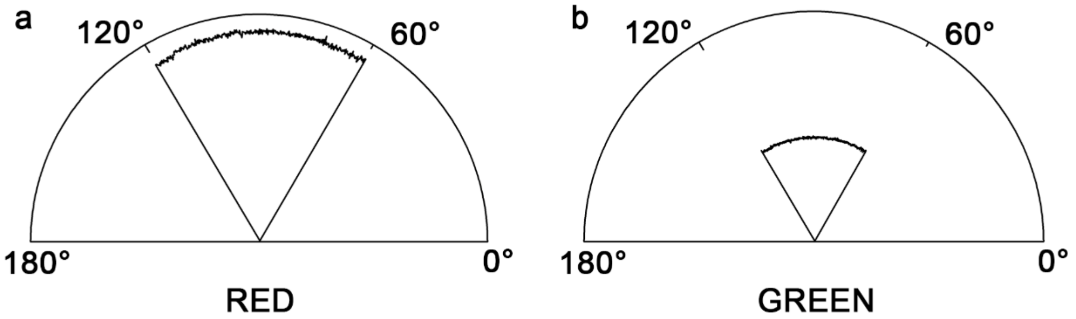

Fig. S2 Radiation pattern under the optical excitation. (**a,b**) Polar plots of scattering intensities measured from the Au nanoantenna with an optical spot size < 1μm. Polar plots of scattering intensities were extracted for the red (**a**) and green **(b**) components. The detected angle range is from 60° to 120°. Scattering intensities are in equivalence in the measured direction for both red and green components.

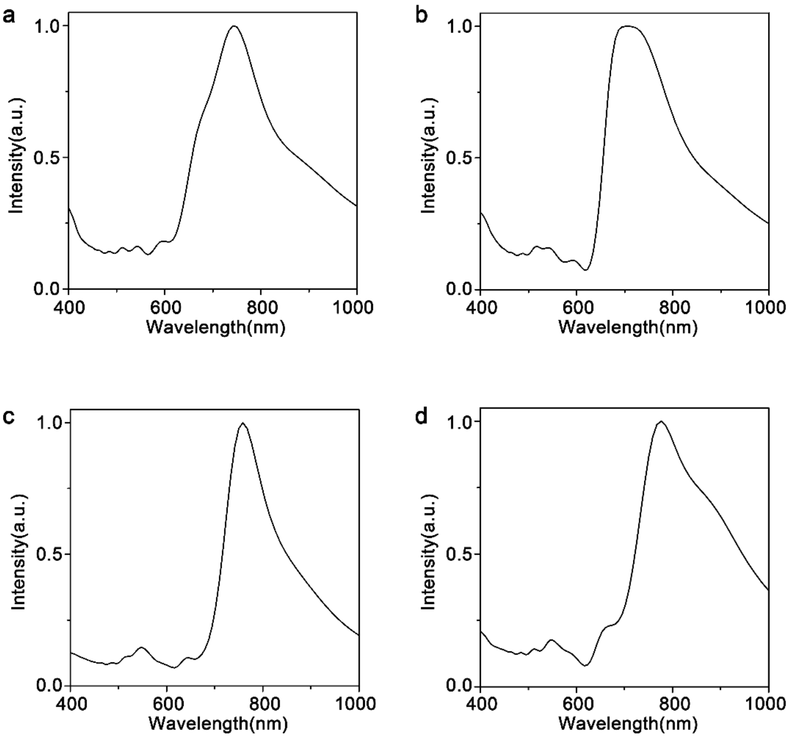

Fig. S3 CL spectra of Au nanoantenna with different sizes. Simulated CL spectra obtained from Au nanoantenna with different sizes under 30 keV electron beam excitation at the upper-right corner. Spectra in panel (**a**) (**b**) (**c**) (**d**) correspond to nanoantenna sizes of 400 nm×90 nm, 400 nm×80 nm, 350 nm×70 nm, and 300 nm×70 nm.

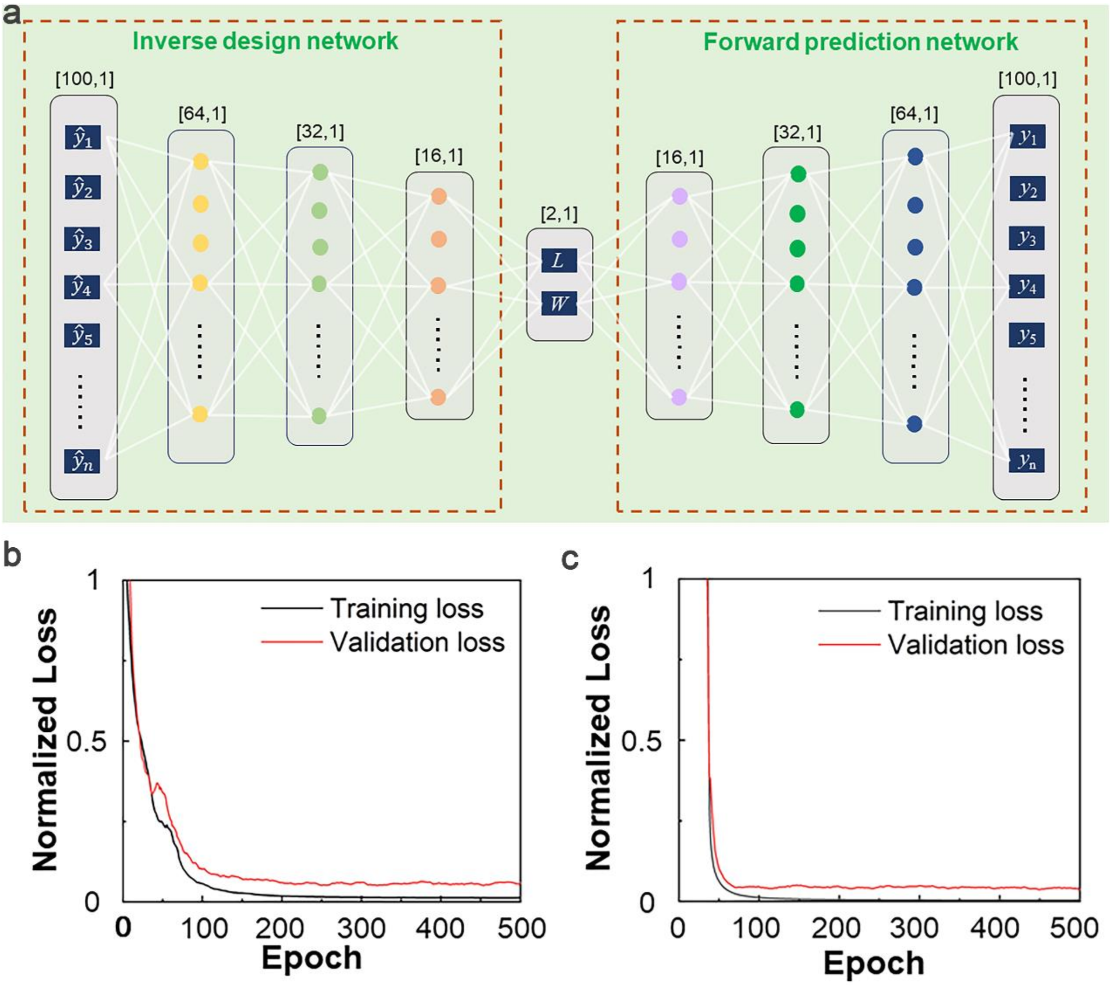

Fig. S4 End-to-end inverse design framework. a The end-to-end inverse design framework consists of an inverse design network and a forward prediction network. Both networks consist of fully connected layers and can accurately characterize the optical properties of meta-atoms. The inverse design network accepts the ideal spectrum $y\hat{}$ as input and predicts the structural parameters L/W. The optical response of L/W is as close as possible to the ideal spectrum $y\hat{}$. The forward prediction network acts as a fast evaluator. It can quickly and accurately predict the actual spectral y corresponding to the structural parameters L/W. It avoids time-consuming simulation verification and enhances the convergence of inverse design networks. After training, the end-to-end inverse design framework can complete the design and evaluate the design results in less than 1 second. **b** Normalized loss of forward prediction network. **c** Normalized loss of inverse design network.

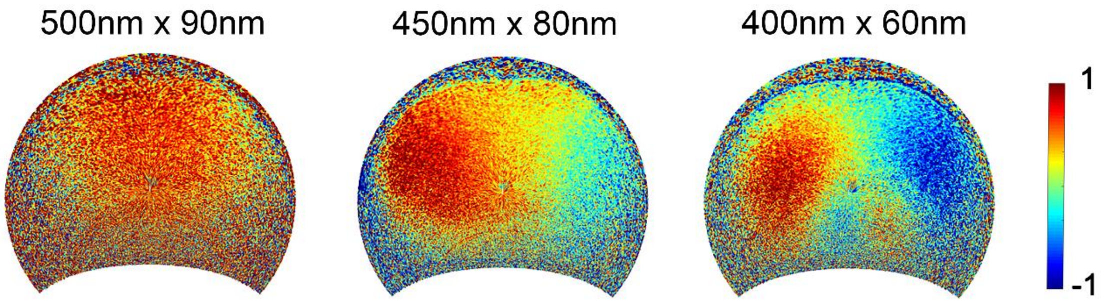

Fig. S5 Experimental angular dichromatic patterns obtained from Au nanoantennas at the size of 500 nm×90 nm, 450 nm×80 nm, 400 nm×60 nm.

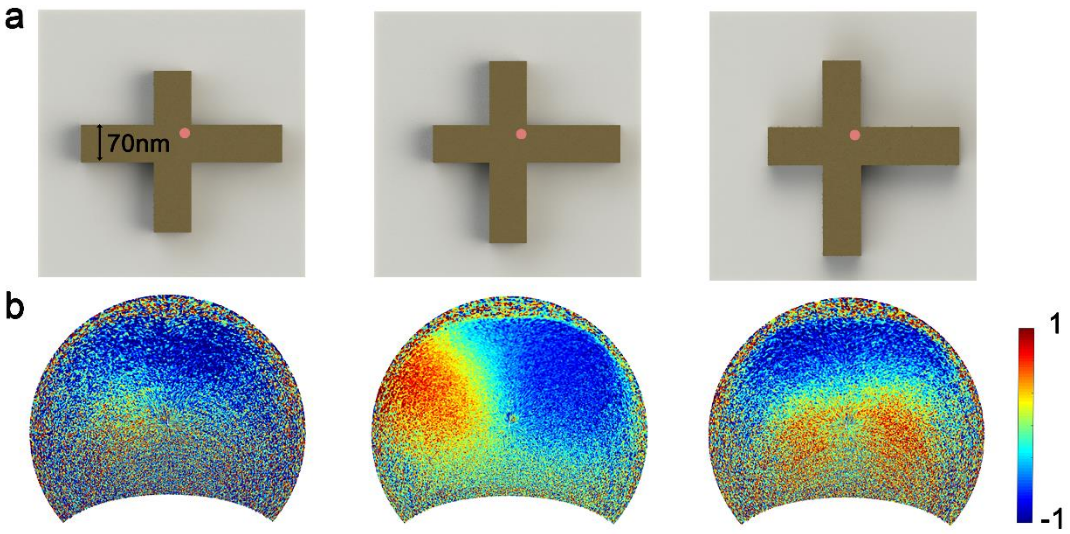

Fig. S6 Experimental angular dichromatic patterns obtained from asymmetric Au nanoantennas at the width of 70 nm. Impinging positions are located at the upper-right corner, which are marked by pink points as shown in **a.** The angular dichromatic patterns corresponding to the asymmetric Au nanoantennas are shown in **b.**

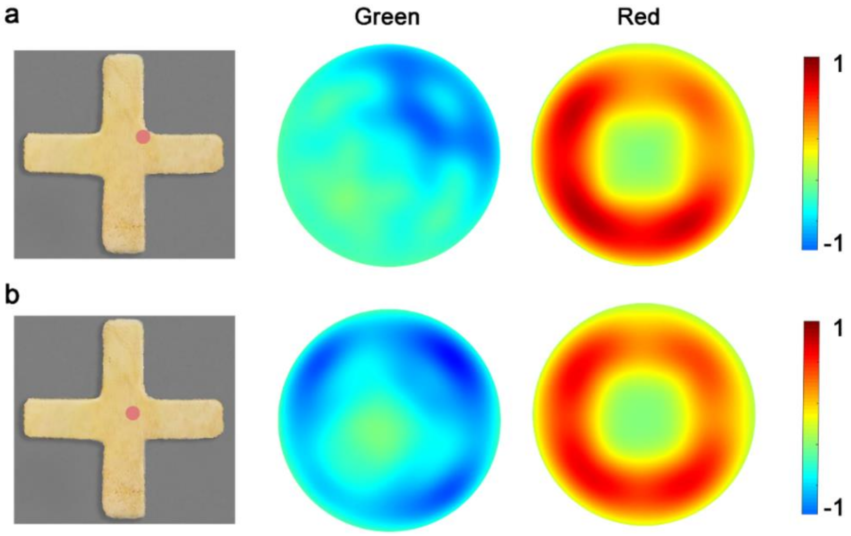

Fig. S7 Simulated angle-resolved responses of the Au antenna with the electron

beam shift. Green and red components of simulated normalized angular patterns obtained from Au nanoantenna with the size of 400 nm×70 nm. Excitation position at the upper-right of the corner **a** and the position that is deviated from the corner **b**.

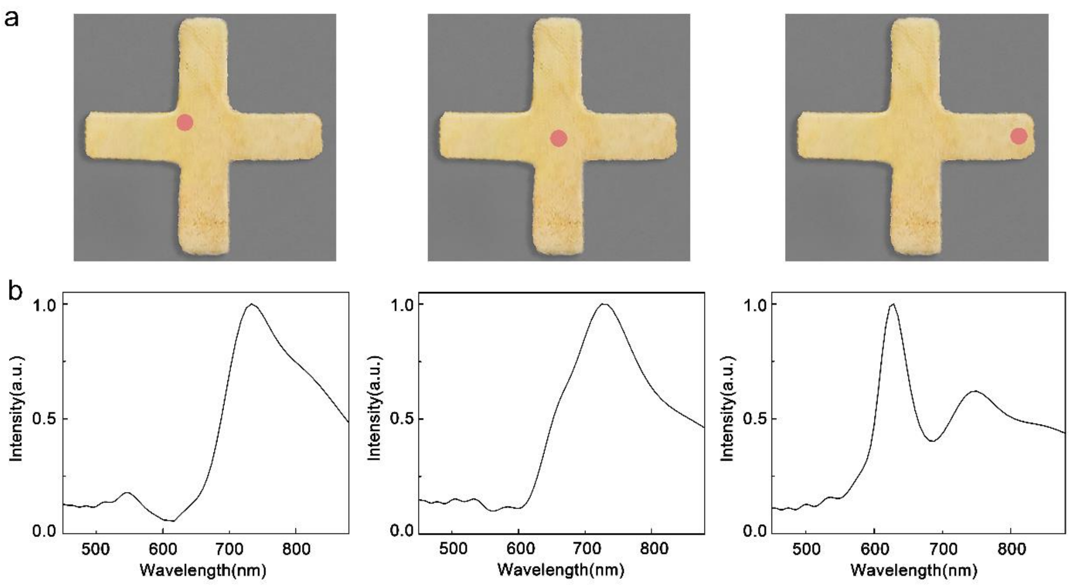

Fig. S8 Measured spectrum of nanoantenna with different excitation positions.

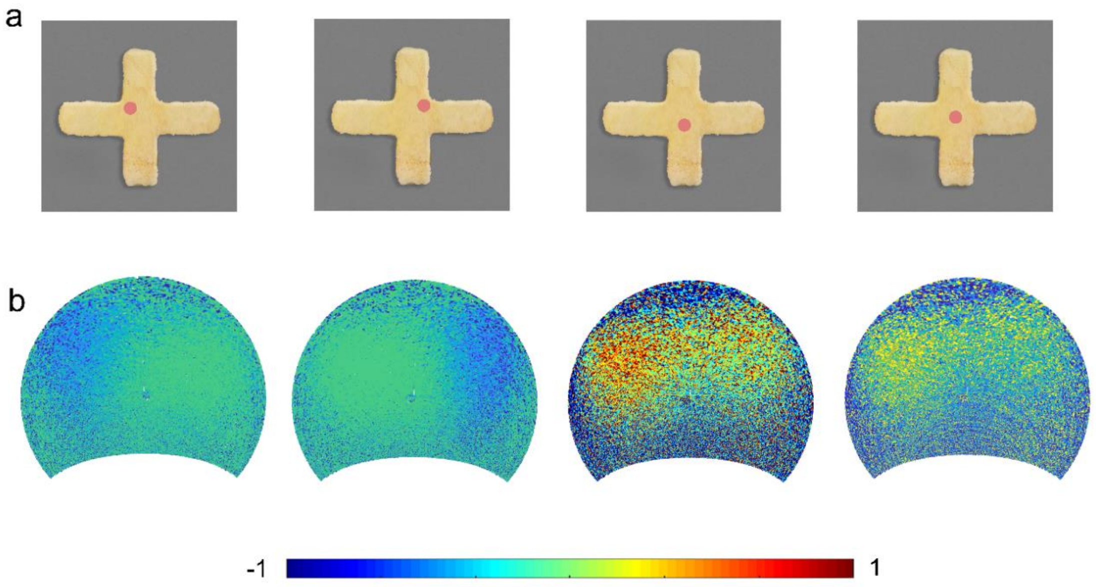

Fig. S9 Manipulation of dichromatic photon momentum. a, b Normalized experimental angular integrated dichromatic patterns obtained from Au nanoantenna at the size of 400 nm×70 nm and 300 nm×70 nm. Stimulation positions are located at the upper-left corner, upper-right corner, center of the lower edge, and center of the nanoantenna, which are marked by red points on the pseudo-colour scanning electron microscopy (SEM) image of the Au nanoantenna as shown in **a**.

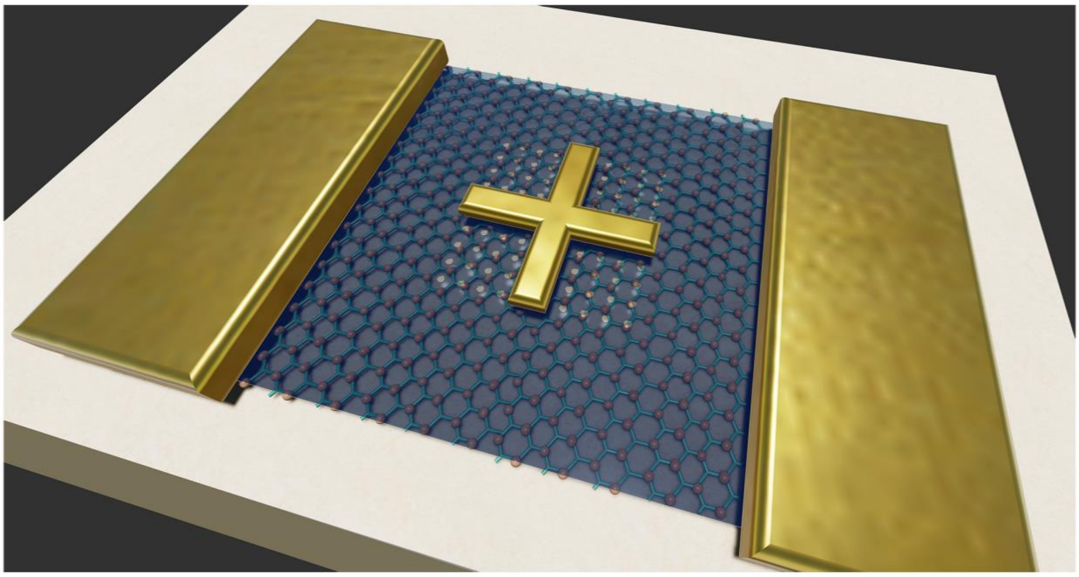

Fig. S10 Schematic of electron-induced CR. Super-aligned carbon nanotube films worked as the filament for electron sources, where the driven voltage is applied through two contacting pads.

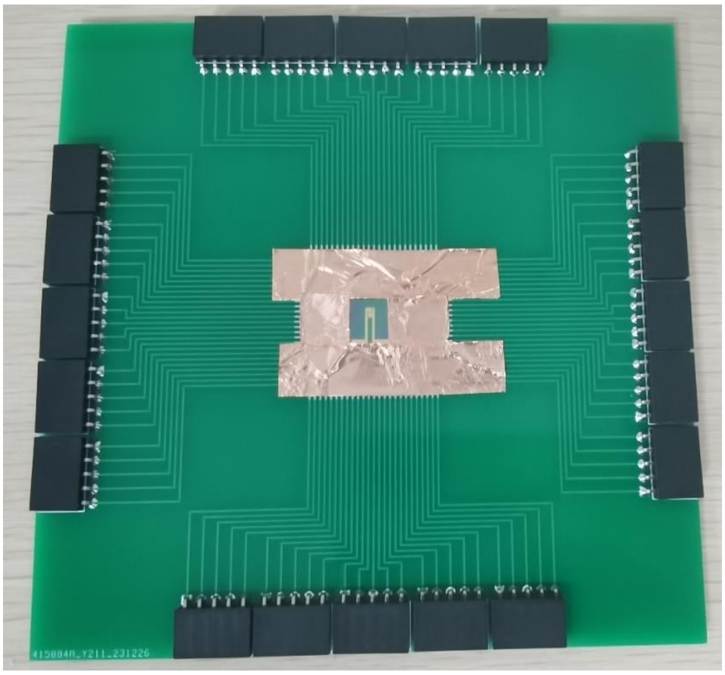

Fig. S11 Image of the colour routers on printed circuit board.

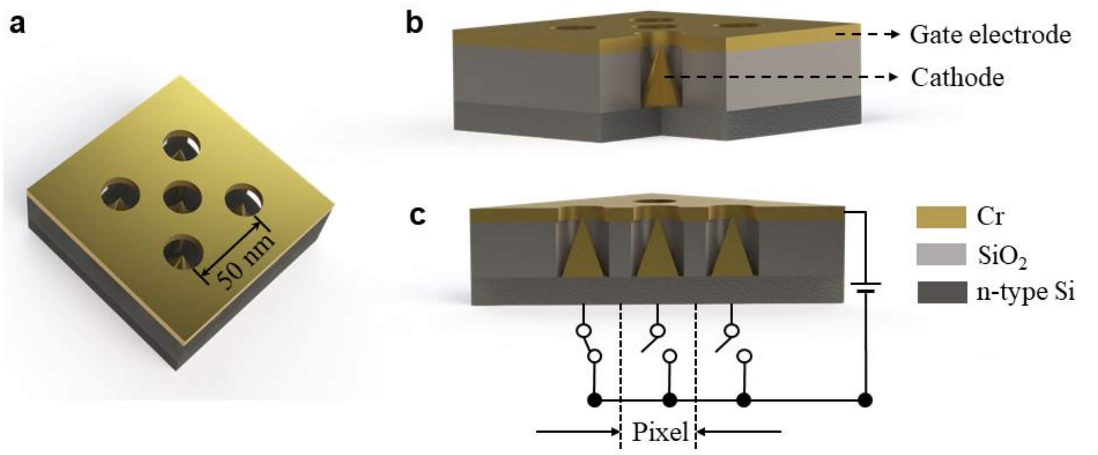

Fig. S12 Schematic of the excitation component beneath the SACNT film. a The excitation component consisting of five emitting cathodes and channel electrodes for the controlled excitation of electron beams. **b** The main view of the excitation component, including the cathodes and gate channels. **c** The pixel unit of the excitation component controlled by the FPGA. The pixel selection circuit is connected to each cathode and independently controls the emitted electron beam.

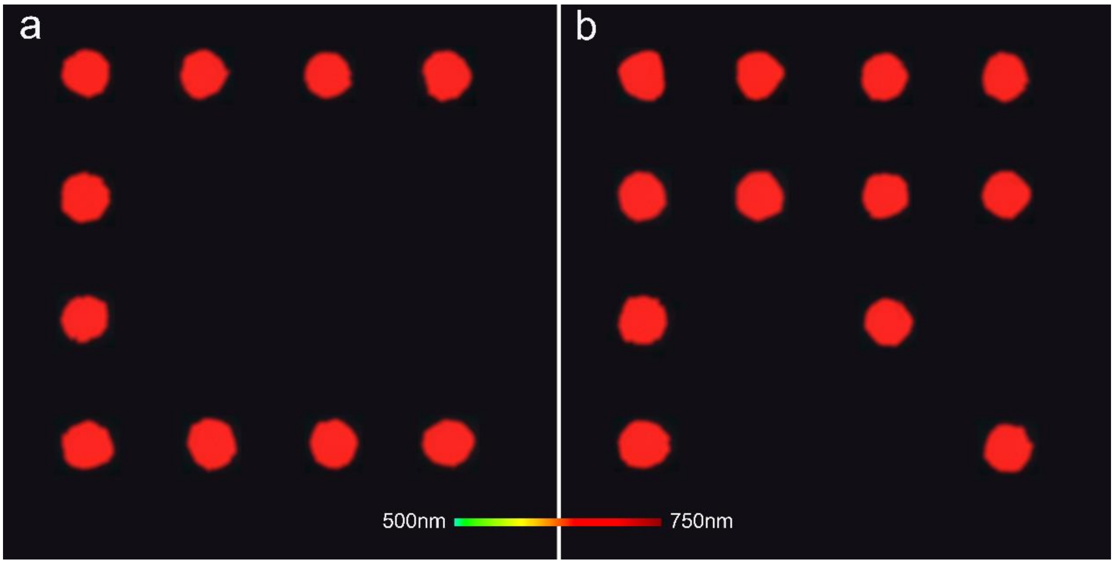

Fig. S13 Two-dimensional display of capital letters. Capital letters “C” and “R” are displayed in **a** and **b**, respectively.

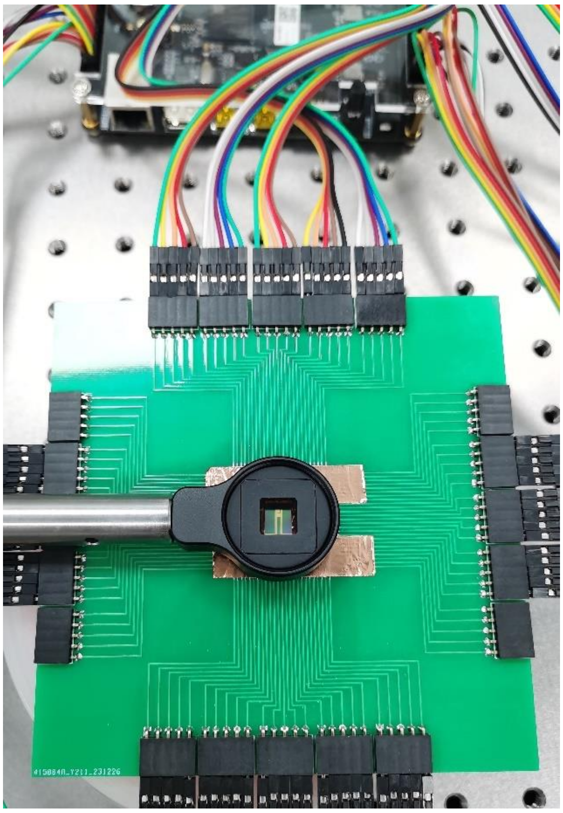

Fig. S14 Fourier read-out of the encryption display device.

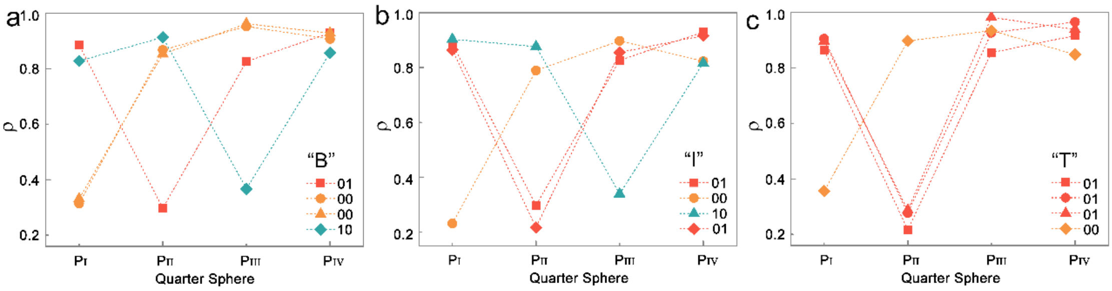

Fig. S15 Intensity ratio analysis of the encryption display device. Intensity ratio (ρ) when the impinging positions shift along a location sequence designed corresponding to ASCII codes, where outputs in panels **a, b,** and **c** correspond to capital letters “B”, “I” and “T”, respectively.

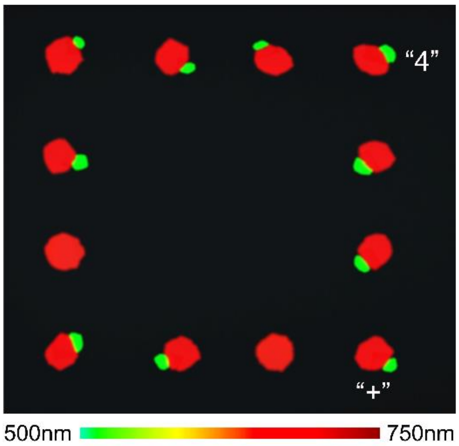

Fig. S16 Fourier read-out of the encryption display device.

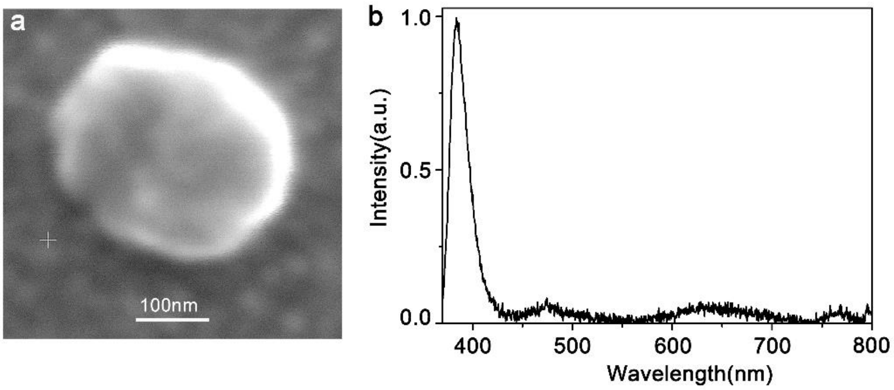

Fig. S17 Al nanoantenna and spectrum characterization. a SEM image of a single Al nanoantenna. **b** Experimental spectrum obtained from Al nanoantenna.

Supplementary Note 1. Far-field angular pattern measurements

In the far-field angular pattern measurements, we measure the angular profile by projecting the beam emanating from the paraboloid mirror onto a 2D CCD camera similar to Fourier microscopy[1-4]. Images are mapped onto angles θ and φ, calculated using the known geometrical properties of the paraboloid and the experimental settings:

$$d_{eff} = d_{pix} n_{mag} b \tag{S1}$$

$$y = y_{pix} d_{eff} \tag{S2}$$

$$z = z_{pix} d_{eff} + d_{foc} \tag{S3}$$

$$r^2 = \sqrt{y^2 + z^2} \tag{S4}$$

$$x = ar^2 - \frac{1}{4a} \tag{S5}$$

$$\theta = \cos^{-1}\left(\frac{z}{\sqrt{x^2 + r^2}}\right) \tag{S6}$$

$$\varphi = \tan^{-1}\left(\frac{y}{x}\right) \tag{S7}$$

where d_{eff} is the effective pixel size which can be calculated from the hardware binning setting in the CCD array b, the individual CCD pixel size d_{pix} (13 μm), and the

demagnification factor of the image by the achromatic lens n_{mag} which corresponds to 2.01 in this case. y_{pix} and z_{pix} are the CCD pixel numbers in the y and z direction respectively, from which the x and z distance from the virtual paraboloid apex can be calculated. Note that one needs to add the focal length of the off-axis paraboloid d_{foc} (0.5 mm) to get the correct z-distance from the apex. Using y and z we can find x, where a is the parabola coefficient (1/10). The solid angle covered per pixel (Ω) which is used to correct the data to photon flux per unit of solid angle is given by

$$\Omega = d_{eff}^2 \frac{2ar^2 - x}{(x^2 + r^2)^{3/2}} \quad \text{(S8)}$$

Here we take the assumption that the pixels are small enough that we can locally regard the mirror surface flat so that we neglect the curvature of the mirror within the projected area of one CCD pixel[5].

Supplementary Note 2. Angle-resolved CL imaging spectroscopy

In the cathodoluminescence (CL) imaging spectroscopy, angular patterns were acquired by a CL detector system, which is mounted on the SEM. The emission was collected by a CMOS to characterize the far-field angular pattern. For detecting specific wavelengths of the CL emission, different bandpass filters were placed in the optical path (Supplementary Fig. S18). The electron beam passing through the pinhole of the parabolic mirror can effectively excite the sample (Supplementary Fig. S20), and the CL emission can be collected by the mirror and is measured using the CMOS array.

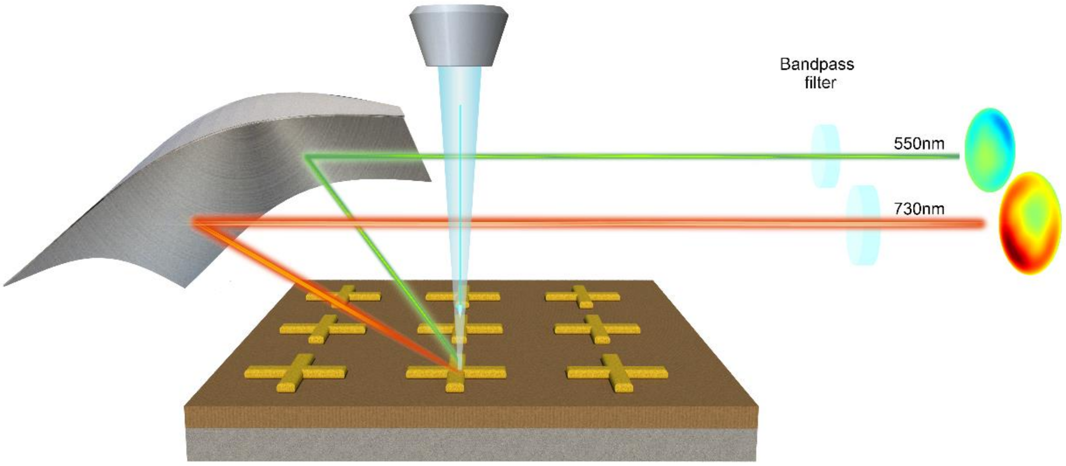

Fig. S18 Angle-resolved CL imaging spectroscopy for measurements of the dichromatic radiation. The bandpass filters are used to extract dichromatic components of CL emission.

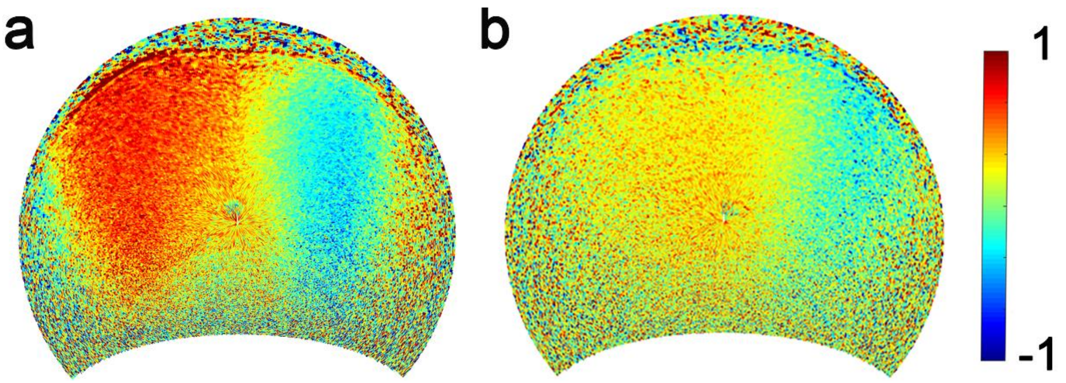

Fig. S19 Experimental angular dichromatic patterns obtained from Au nanoantennas with filters of **a** 700 nm and 580 nm (50 nm bandwidth), **b**. 730 nm and 550 nm (10 nm bandwidth).

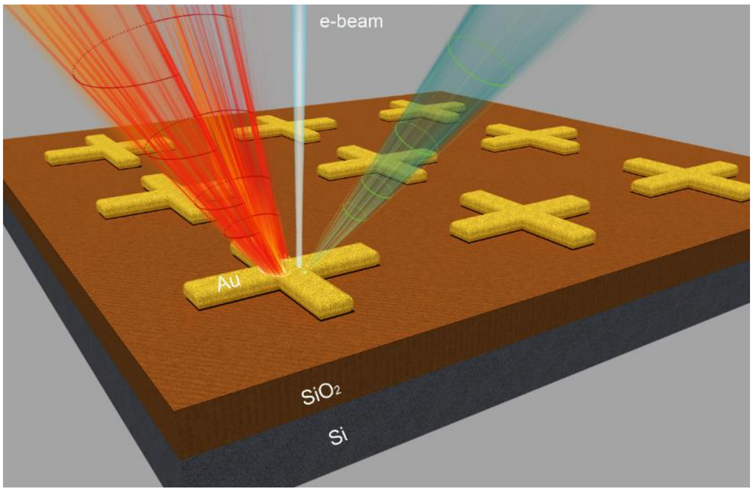

Fig. S20 Schematic illustration of electron-induced CRE with angle-resolved CL detection. Symmetrical Au nanoantenna under electron beam excitation at the corner generates asymmetrical dichromatic dispersion radiation.

Supplementary Note 3. Read-out rules for the encrypted display

In the encrypted display of 4x4 units, the light and dark states are shown in solid circles and hollow circles in supplementary Fig. S21, respectively. An 8-digit sequence of four units in a row/column corresponds to a character in ASCII (American Standard Code for Information Interchange) codes. The series number of codes from 1 to 8 corresponds to the sequence number of rows and columns in Supplementary Fig. S21. The read-out orders of the encoding sequence are shown in the arrow, where valid codes need four units in the sequence all in the alight state as codes in series number 1-2, 4-5. When the dark state exists in the unit, invalid codes are read out as codes in series number 3, 6-8.

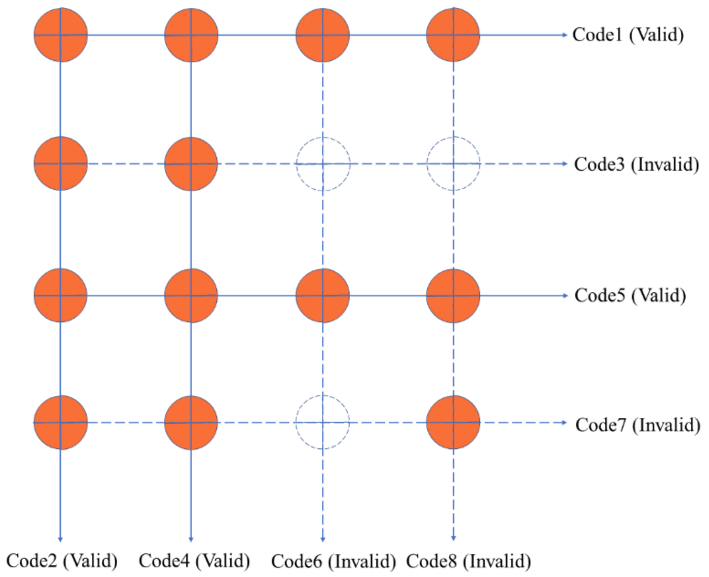

Fig. S21 Read-out rules for the encrypted display. Codes in series number 1-8 are read-out in order according to the proposed rules. Valid codes represent encrypted information, while invalid codes possess no information.

Reference

1. Lee, K. G. *et al*. A planar dielectric antenna for directional single-photon emission andnear-unity collection efficiency. *Nat. Photonics* **5**, 166 (2011).
2. Curto, A. G. *et al*. Unidirectional emission of a quantum dot coupled to a nanoantenna. *Science* **329**, 930 (2010).
3. Sersic, I., Tuambilangana, C. & Koenderink, A. F. Fourier microscopy of single plasmonic scatterers. *New. J. Phys.* **13**, 083019 (2011).
4. Lieb, M., Zavislan, J. & Novotny, L. Single-molecule orientations determined by direct emission pattern imaging. *J. Opt. Soc. Am. B* **21**, 1210 (2004)
5. Takeuchi, K. & Yamamoto, N. Visualization of surface plasmon polariton waves in two-dimensional plasmonic crystal by cathodoluminescence. *Opt. Express* **19**, 12365 (2011).
